# Use of a Simply Modified Drainage Catheter for Peritoneal Dialysis Treatment of Acute Renal Failure Associated With Cardiac Surgery in Infants

**DOI:** 10.1097/MD.0000000000000077

**Published:** 2014-09-19

**Authors:** Qiang Chen, Hua Cao, Yun-Nan Hu, Liang-Wan Chen, Jia-jun He

**Affiliations:** Department of Cardiovascular Surgery (QC, HC, Y-nH), Union Hospital, Fujian Medical University, Fuzhou 350001, P.R. China.

## Abstract

Acute renal failure (ARF) is a common complication in infants who undergo cardiac surgery in the intensive care unit. We report on a modified drainage catheter used in peritoneal dialysis (PD) for the treatment of ARF associated with cardiac surgery in infants.

Thirty-nine infants with congenital heart disease undergoing cardiac surgery who developed ARF at our center between January 2009 and January 2012 were assessed. A modified drainage catheter for PD was used in these infants. Their demographic, clinical, and surgical data were analyzed.

Thirty infants with ARF were cured by PD, and the other 9 died in the first 48 hours because of the severity of the acute cardiac dysfunction. All these infants were dependent upon mechanical ventilation during the postoperative period and used vasoactive drugs. In the survival group, the interval between the procedure and initiation of PD was 13.6 ± 6.5 (range, 6–30) hours. PD duration was 3.9 ± 0.9 (3–6) days. Minor complications were encountered in some patients (asymptomatic hypokalemia, hyperglycemia, and thrombocytopenia). These complications were readily treated by drugs or resolved spontaneously. Hemodynamics, cardiac function, and renal function improved significantly during PD.

These data suggest that PD using a modified drainage catheter for ARF after cardiac surgery in infants is safe, feasible, inexpensive, and yields good results.

## INTRODUCTION

Acute renal failure (ARF) is a complication diagnosed in patients undergoing cardiac surgery for the correction of congenital heart disease and is associated with an increased prevalence of mortality.^1^–4 Despite considerable progress in cardiac surgery and intensive care procedures, the prevalence of ARF in children with normal healthy kidneys undergoing open heart surgery has been reported to be 2% to 8% with a high prevalence of mortality. Most studies have not classified the etiology and/or pathogenesis of this type of ARF.

Risk factors that have been suggested for the development of ARF include young age, complex cardiac lesions, prolonged cardiopulmonary bypass (CPB) time, and low cardiac output state after open heart surgery.^5^–7 Fluid restriction, diuretics, and inotropic agents are the initial therapeutic strategies for mild renal dysfunction and low cardiac output syndrome. More severe cases require slow and continuous removal of fluid by hemofiltration or peritoneal dialysis (PD). Compared with hemofiltration, PD enables the establishment of vascular access, avoidance of systemic anticoagulation, and decreased associated risks of ischemic and embolic complications.^8^–10 The role of PD and indications in postoperative therapy and the technical details of applying it (eg, optimal timing of PD, contents of the dialysate solution, and indwelling volume and its complications) are controversial.

The aim of the present study was to evaluate the safety and feasibility of a modified drainage catheter for PD used in infants with ARF after open heart surgery.

## MATERIALS AND METHODS

The study protocol was approved by the ethics committee of Fujian Medical University and adhered to the tenets of the Declaration of Helsinki. Written informed consent was obtained from the parents of all the patients included in this study.

### Patients

The hospital records of 39 patients who underwent heart surgery with CPB for congenital heart disease between January 2009 and January 2012 were reviewed. Patients were aged 1 to 11 months (mean ± standard deviation, 4.7 ± 2.6 months). Their weights ranged from 3.5 to 6 kg (4.5 ± 0.7 kg). All patients needed inotropic support and mechanical ventilation. They developed ARF and needed PD treatment.

We divided patients who underwent PD into 2 groups depending on outcome. In group I, 30 infants survived after PD, and in group II, 9 children died after PD (Table 1). Age, sex, weight, CPB time, aortic cross-clamp time, preexisting renal disease, beginning of PD, details of lesion, and type of cardiac surgery undertaken were recorded (Table 2).

**TABLE 1:**
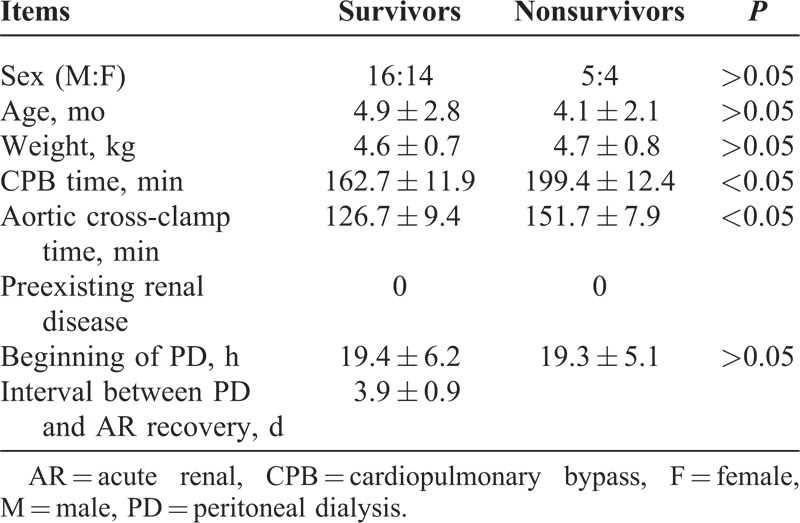
Clinical Data Collection From All Infants With Cardiac Surgery Undergoing PD

**TABLE 2:**
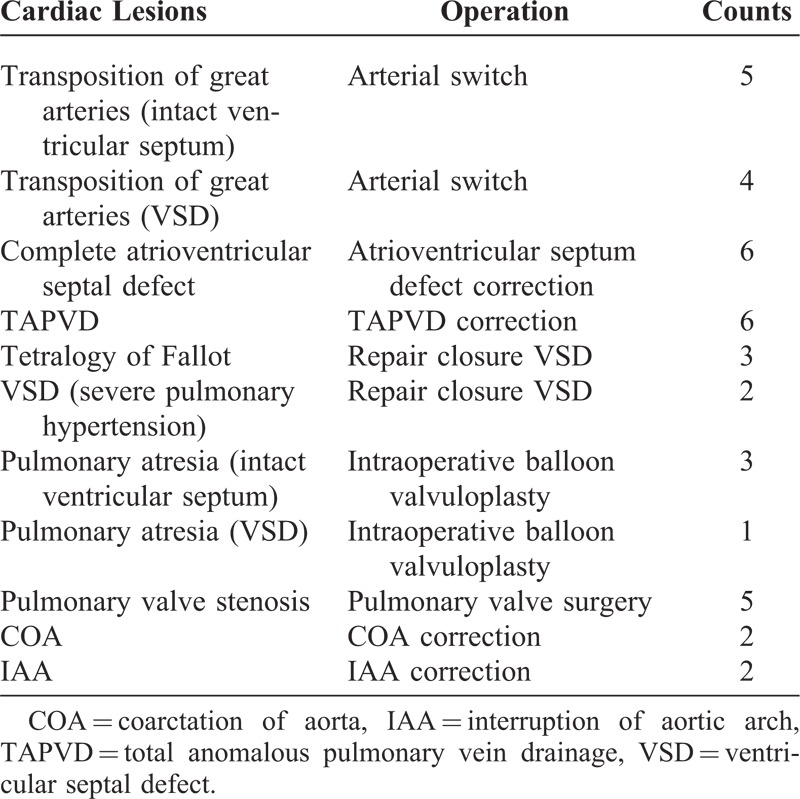
Details of Lesion and Operation

ARF was defined as urine output <0.5 mL/kg/h for >4 hours; unresponsive to aggressive diuretic therapy, adjustment in fluid therapy, and optimization of inotropic support; and preoperative creatinine level that at least doubles in patients aged >8 weeks, is >75 mmol/L for patients aged <8 weeks, or any combination thereof.

### Peritoneal Dialysis

PD is the first-line treatment for postoperative ARF at our institution because of its relatively easy employment and severe complications are rarely associated with PD. The indications for the initiation of PD at our institution are oliguria for >4 hours despite medication interventions, and, in the absence of established oliguria, increased creatinine level associated with one of the following: clinical signs of fluid overload, severe edema, hyperkalemia (>5.5 mmol/L), hyperphosphatemia (>4 mmol/L), persistent metabolic acidosis, or low cardiac output syndrome. Metabolic acidosis was considered persistent if it failed to be corrected by at least 2 boluses of intravenous sodium bicarbonate infusion as well as adjustment of fluid status and inotropic support. Low cardiac output syndrome was defined as a mean arterial pressure (MAP) of <60 mm Hg on 3 or more separate readings for infants, and requirement of 2 or more inotropic agents. Indications for stopping PD were the return of sufficient urine output to maintain or achieve negative fluid balance as well as normalization of serum electrolytes and acid–base status.

A modified homemade silicone rubber peritoneal catheter was inserted surgically through a left-sided paraumbilical approach during the primary surgical procedure (especially in the case of long CPB time, long circulatory arrest time, low cardiac output, or delayed closure of sternotomy) or in the intensive care unit if postoperative hemodynamic and renal complications occurred. Placement of the peritoneal catheter could be prophylactic and therapeutic, but initiation of PD was in accordance with the indications described above.

The surgical procedure comprised opening of the peritoneum under the abdominal oblique muscle through extraperitoneal fat at the left lower quadrant abdominal for 1 to 2 cm. Artery forceps were passed through the peritoneal opening made at the surgical incision and brought out through the peritoneum, tunneling the PD catheter subcutaneously and then passing it through the abdominal wall with a purse string around the opening. The PD catheter was connected to a closed system for peritoneal drainage using 2 tee switches that were connected separately to the dialysate solutions and the 50 mL cylinder. Through the use of this cylinder, we could calculate the volume of the dialysate solutions. Through the use of the 2 tee switches, we could pass the dialysate solutions in and out. Care was taken in placing the purse string in the peritoneal incision; a loose stitch may allow dialysis fluid to leak into the skin and a “snug” purse string can kink the catheter.^11^

We used 10-F and 30-cm silicone dialysis catheters, which we used for the incision drainage, as opposed to the Tenckhoff PD Catheter (Quinton; Tyco Health Care Group, Mansfield, MA). The advantages of this catheter are very low cost, smaller diameter, and ease of accessibility. Then, 10 to 15 small bores were made in the front of the catheter, which enabled the creation of adequate individual draining holes as well as a longer length of draining surface.

The dialysate solutions used were standard commercial preparations (Dianeal PD-2; Baxter Healthcare, Guangzhou, China) with a dextrose concentration of 1.5%, 2.5%, or 4.25%. In cases of clinically suspected infection, the dialysate was examined by microbiological means. An antibiotic was added if there was clinical evidence of sepsis or peritonitis. PD was started with a dwell volume of 10 mL/kg, a fill time of 50 minutes, and a drainage time of 10 minutes. According to the targeted fluid balance, the dextrose concentration varied from 1.5% to 4.25% and the dwell time varied from 1 to 2 hours and, if necessary, the dwell time was adjusted. The dialysate bags were changed every 24 hour. The frequency of peritoneal exchange, the dextrose concentration, dwell time, and drainage time varied depending on patient needs that changed regularly according to clinical and biochemical status. Serum levels of albumin and electrolytes were monitored regularly, and intravenous infusions of albumin and electrolytes were given as necessary.

### Statistical Analyses

Continuous data are given as the mean ± standard deviation and range. Clinical parameters were compared with the *t* test of independent samples. Nominal variables were compared using Fisher exact test. *P* < 0.05 was considered significant.

## RESULTS

Thirty infants with ARF were cured by PD, but the other 9 subjects died in the first 48 hours because of the severity of the acute cardiac dysfunction. All these infants were dependent upon mechanical ventilation during the postoperative period and used vasoactive drugs. In the survival group, the interval between the procedure and initiation of PD was 13.6 ± 6.5 (range, 6–30) hours. The duration of PD was 3.9 ± 0.9 (3–6) days. Minor complications were encountered in the survival group (asymptomatic hypokalemia, hyperglycemia, and thrombocytopenia). These complications were treated by drugs or resolved spontaneously (Table 3).

**TABLE 3:**
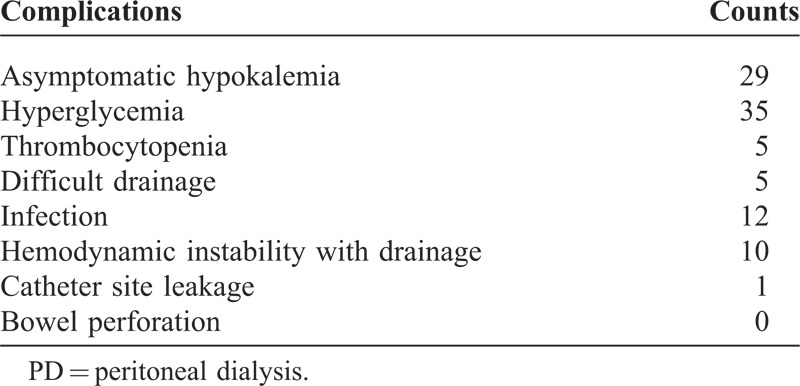
Complication of PD in the Survival Group

In the survival group, the following data were recorded prospectively: heart rate (HR), MAP, central venous pressure (CVP), use of inotropic drugs, blood sugar, serum creatinine, blood urea nitrogen (BUN), potassium, sodium, calcium, urine output, fluid input, PD ultrafiltration, fluid balance, and arterial blood gas parameters (Table 4). For each of the recorded variables, 3 values were retrieved daily and the mean calculated. Some measurements were taken before PD and 1, 3, and 5 days after PD.

**TABLE 4:**
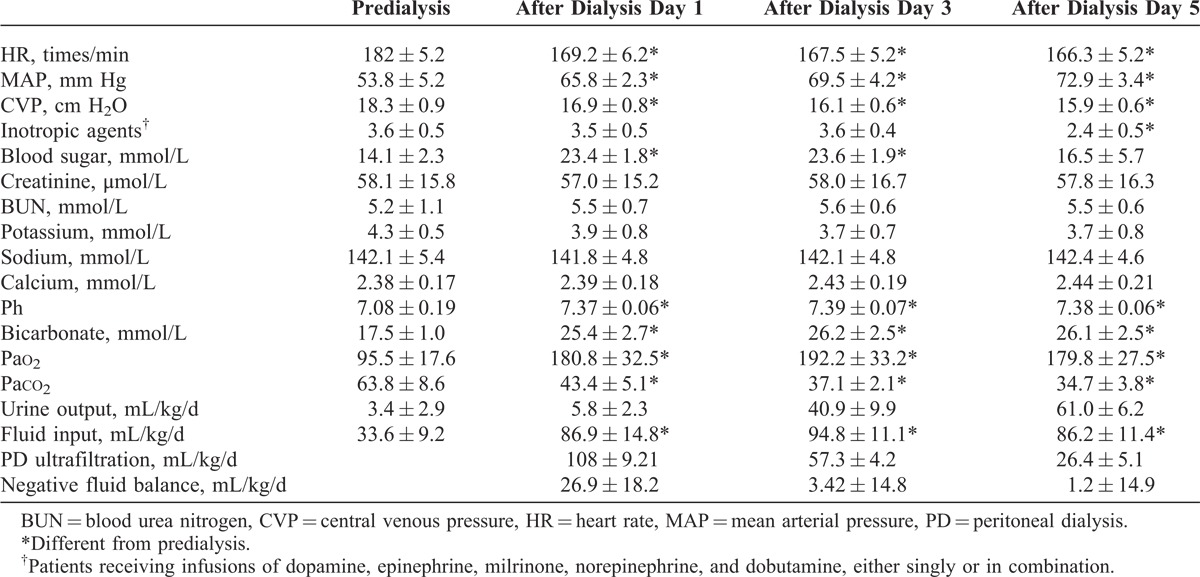
Clinical Data Collection From the Survivors Undergoing PD

Elapsed time after open heart surgery to the institution of PD, duration of PD, and complications were investigated. The efficacy of dialysis was measured by fluid balance and time-to-correction of ARF. Nephrotoxic medications were avoided, or the dose was adapted to renal function and, if possible, was checked by serum levels. Table 4 demonstrates the comparison of clinical data of all survivors before and after PD: evidence confirmed that hemodynamics, cardiac function, and renal function improved appreciably during PD.

In the survivors, PD was, in general, stopped when oliguria or anuria resolved. Recovery from ARF was defined as reduction of the serum level of creatinine to baseline levels after cessation of PD. Death and cause of death were noted. Nine infants died in the first 48 hours because of the severity of the acute cardiac dysfunction.

## DISCUSSION

Congenital heart disease often leads babies to have repeated colds and congestive heart failure. Timely surgery is the only effective treatment for these patients. Because the kidneys of child patients are under relatively low perfusion pressure during the extracorporeal circulation, surgical trauma, low temperature, and intraoperative and postoperative infusion of saved blood can cause systemic inflammatory responses that often lead to acute postoperative renal insufficiency and even renal failure.^12–14^ During ARF, the retention of sodium and water directly increases cardiac preload and hinders the recovery of cardiac function. Prolonged oliguria or anuria result in disturbances of water and electrolytes and aggravate pulmonary edema, which lowers the gas exchange of alveoli and induces respiratory insufficiency. Thus, serum creatinine is elevated continuously and followed by hyperkalemia and acidosis, which is life-threatening. Therefore, timely treatment of postoperative renal dysfunction of these children is particularly important.^15^

As early as the 1950s, there was a succession of articles focusing on hemodialysis in children.^16^ However, the studies were mainly in the early postoperative period with incompletely restored cardiac function, unstable hemodynamics, poor vascular access, limited fluid volume, and poorly controlled ultrafiltration in hemodialysis. Hemodialysis necessitates continuous anticoagulation that is not beneficial for hemostasis in early postoperative wounds. Thus, hemodialysis in young children is, to a large extent, restricted. Therefore, in the present study, emergency PD was used to treat the postoperative acute renal insufficiency of babies after cardiac surgery.

PD in babies has several advantages. The peritoneum of babies has a 2-fold larger area than that of adults, and PD can be implemented more readily in babies than in older children and adults. In general, PD is relatively simple and does not require complex equipment. Its cost is low, compliance in children and acceptance by parents is high, systemic anticoagulation or prefilled blood products are not needed, and it is relatively safe. The efficiency of dialysis is high, and the elimination effect on medium-sized molecular substances such as urea and nitrogen, as well as small molecules such as creatinine, phosphorus, and potassium, is good.^17–19^ Therefore, in the present study, PD was chosen as the main treatment of ARF in babies after CPB.

PD in babies can result in problems related to catheter implantation. Some complications of PD are associated with surgical incision of the planting pipe, which can cause improper positioning or displacement of the catheter.^20–22^ Therefore, an experienced surgeon is needed for catheter implantation. We used a simple homemade catheter and ensured that catheter implantation in the pelvic cavity was based on the anatomical characteristics of the omentum. The incision was double stitched, and the external oblique fascia was sutured to lessen the chances of catheter displacement. The peritoneum and abdominal muscles can be partially and continuously sutured to greatly reduce the chance of leakage. One patient in this group had leakage, but it resolved after the application of bandages. The PD tube is drawn out from the hole in the outlet to minimize contamination of the original incision. According to our experience, the simple homemade catheter can be used with the same clinical effect if a specific dialysis tube for infants is not available.

Keeping the dialysis tube free of obstructions is crucial to PD. The dialysis tube should be carefully fixed to prevent stretching, folding, and obstruction. Floccules were found in the drainage tubes of 5 children and were believed to be fibrin plugs or omental adhesions. Catheter drainage was significantly improved after flushing and addition of heparin to the dialysate. If the difference between the outlet and input volumes of fluid is significant, the body position changes, and abdominal massage and pipe extrusion are ineffective, then sucking hard with a syringe to prevent omentum inhalation into the micropores of the dialysis tube is not helpful. In this case, repositioning of the catheter is the best choice.

When choosing PD, we advocate implantation of a dialysis tube and provide dialysis treatment as soon as possible for high-risk patients if early postoperative renal dysfunction is expected.^23^ The frequency of PD is tailored according to the specific needs of the patient; the velocity and amount of input liquid should be controlled. In our experience, the initial regimen is 10 mL/(kg × time) with a retention time of 50 minutes followed by opening for 10 minutes. PD is done for 1 hour in the early period. According to the recovery of urine, the period of PD is extended gradually to 2, 4, and 8 hours and stopped until renal function recovers to normal levels. We chose a dialysate concentration of 4.25% at the peak of edema, which was then changed gradually to 2.5% or 1.5% according to the recovery of renal function.

According to the comparison between the death group and survival group, the operation time of the death group was significantly longer than that of the survival group. This finding was related to the complexity of the illnesses in the death group; because of the extension of operation time, refractory postoperative heart failure and kidney failure occurred, but these disorders cannot be cured with PD even if PD is instigated early. In the survival group, the effects of PD were satisfactory. The hemodynamics stabilized gradually after the start of PD, lung function improved, urine output increased gradually, the duration of dialysis was shortened, and renal function was completely repaired. During PD, the effect of dialysis should be evaluated by careful observation of the color, nature, and transparency of the dialysate. To provide a reliable basis for the evaluation of dialysis, we recorded the volume of dialysate, urine output, HR, blood pressure, CVP, and temperature changes; periodically reviewed levels of electrolytes, BUN, and creatinine; carried out routine examination of blood and urine; and determined colloid osmotic pressure and protein content in plasma. Particular attention should be paid to liquid exclusion of dialysis patients with good effects, which could cause hypokalemia and hypocalcemia; thus, intravenous potassium and calcium should be done promptly. When the amount of urine was increased significantly compared with that before PD and the output of dialysate was larger than the input, the effect of PD was assumed to be good.

We found that, in most cases, blood sugar was persistently high and countering this effect with insulin during PD was difficult, but blood sugar levels returned to normal after PD was stopped. In death cases, hypoglycemia was very difficult to correct during PD. In this group, significantly enhanced creatinine levels were not observed because the preoperative renal function of most of the patients was normal; renal failure was associated mainly with surgery. In addition, symptoms were centered on the retention of sodium and water as well as edema, which did not affect the change in creatinine levels in the short term.

During PD, attention should be paid to certain aspects. First, strict aseptic technique should be implemented with careful observation of the dialysate and incision wound. In the present study, a closed dialysate input–output system was chosen, which was more conducive to the aseptic technique. Second, the input velocity of the dialysate should not be too fast; it should finish in 5 to 10 minutes to prevent a sudden jump in abdominal pressure, which would affect respiratory and circulatory functions. Similarly, the tapping velocity should not be too fast; otherwise, it may lead to hypotension and unstable hemodynamics. Third, strict control of the dialysate temperature is important. A relatively high or low temperature can cause discomfort and even affect dialysis. We used an insulation blanket to preheat the dialysate to normal body temperature, and this temperature was maintained. Fourth, nutritional support and basic nursing care should be enhanced. During PD and before the recovery of gastrointestinal function, parenteral nutrition should ensure the energy and protein requirements of the body. If the circulation is not stable, a water bag should be placed under the back; after the circulation has stabilized, attention should be paid to changing the body position to prevent pressure sores.

The prevalence of mortality of renal replacement patients after CPB has been reported to be high (between 14% and 82%). We found the mortality to be ≤23.1%. Apart from the fact that the risk of cardiac surgery in younger patients is high and complications numerous, this high prevalence could be related to the time of PD. For instance, for the 5 dead infants, PD was started after postoperative anuria and diuresis was indicated. For critically ill infants with a long operation time and potential renal failure, prophylactic PD could improve the prognosis.^23–25^ Certainly, the effect of PD for infants, optimal timing, PD regimen, and even the length of the dialysis tube needs further investigation.

The present study suggested that PD can cure or alleviate ARF after cardiac surgery in infants to some extent. However, if the increase in urine output is not significant, renal function is not improved, or the disease is worsened after a period of PD for infants with postoperative ARF, a switch to hemodialysis is indicated.
